# Grand Challenges: Integrating Mental Health Care into the Non-Communicable Disease Agenda

**DOI:** 10.1371/journal.pmed.1001443

**Published:** 2013-05-14

**Authors:** Victoria K. Ngo, Adolfo Rubinstein, Vijay Ganju, Pamela Kanellis, Nasser Loza, Cristina Rabadan-Diehl, Abdallah S. Daar

**Affiliations:** 1 RAND Corporation, Santa Monica, California, United States of America; 2 Institute for Clinical Effectiveness and Health Policy, University of Buenos Aires, Buenos Aires, Argentina; 3 Behavioral Health Knowledge Management, Austin, Texas, United States of America; 4 Grand Challenges Canada, Toronto, Ontario, Canada; 5 Behman Psychiatric Hospital, Cairo, Egypt; 6 National Heart, Lung, and Blood Institute, Bethesda, Maryland, United States of America; 7 Dalla Lana School of Public Health and Dept. of Surgery, University of Toronto, Toronto, Ontario, Canada; 8 Stellenbosch Institute for Advanced Study (STIAS), Wallenberg Research Centre at Stellenbosch University, Stellenbosch, South Africa

## Abstract

In the third article of a five-part series providing a global perspective on integrating mental health, Victoria Ngo and colleagues discuss the benefits and requirements of collaborative care models, where non-communicable disease and mental health care are integrated and provided in the primary care setting.

*Please see later in the article for the Editors' Summary*

**Summary Points**

- Non-communicable chronic diseases (NCD) and mental disorders each constitute a large portion of the worldwide health care burden, and they often occur together.
- Collaborative care models, where NCD care and mental health care are integrated and provided in the primary care setting, are effective for patients, strengthen health care service systems, and reduce costs.
- Using lay health workers to supplement the services provided by mental health specialists, physicians, and nurses can extend services to more patients, but raises challenges related to training and coordination.
- Implementation of collaborative care models and scale up of successful models will be enhanced by tapping local knowledge of social, political, cultural, and health system nuances.
- Collaborative care approaches that integrate services for NCD and mental health conditions require investments in human resources, services, and additional research.
- This is the third in a series of five articles providing a global perspective on integrating mental health.

*This is one article in a five-part series providing a global perspective on integrating mental health.*

## Introduction

As countries develop and progress, health priorities must expand beyond eradication of communicable diseases to include control of non-communicable chronic diseases (NCD). Four primary NCD – cardiovascular disease (mainly heart disease and stroke), type 2 diabetes, some cancers, and chronic respiratory diseases — henceforth referred to as “physical” NCD — are responsible for 35 million deaths annually. They are the leading cause of mortality in the world, much of which is premature and avoidable. Nearly 80% of NCD deaths occur in low- and middle-income countries [1]. Over the last 20 years, the burden of disease, i.e., the impact of NCD worldwide as measured by morbidity and mortality, rose from 47% to 54% [2]. An aging population, longer life expectancies, population growth, urbanization, and globalization of risk factors have made NCD a threat to worldwide development and economic growth and an urgent global health priority.

This article, the third in a series of five, argues that mental health care should be integrated into the NCD agenda, reviews the evidence for models of integration in high- and low-income countries, identifies the challenges and opportunities for addressing the rising burden of mental health and NCD, and recommends strategies to advance a more integrated agenda.

## Evidence for Integration

### The Strong Connection between Mental Illness and NCD

The burden of mental illness has been underestimated, in part, because the links between mental health and other health conditions are not well understood. As the population grows and ages, more individuals live longer with physical NCD and mental illness [2]. These chronic conditions are related in complex ways. Major modifiable risk factors for NCD, such as poor diet, physical inactivity, tobacco use, and harmful alcohol use, are exacerbated by poor mental health. Mental illness is a risk factor for NCD; its presence increases the chance that an individual will also suffer from one or more chronic illnesses. In addition, individuals with mental health conditions are less likely to seek help for NCD and symptoms may affect adherence to treatment as well as prognosis [3],[4].

Depression and disorders related to alcohol use predict the onset, progression, management, and level of disability associated with the NCD [5]–[7]. The prevalence of major depression is consistently higher for persons with physical illnesses than for those without these disorders; e.g., 29% with hypertension, 22% with myocardial infarction, 27% with diabetes, and 33% with cancer [8]. The odds of noncompliance with medical treatment regimens are three times greater for depressed patients compared with non-depressed patients [9]. Health-related quality of life is significantly lower for depressed patients than for patients with asthma, arthritis, and diabetes [6].

Alcohol use is causally linked to eight different cancers, and the risk of developing these cancers increases with increased rate of consumption. Similarly, alcohol use is related to many adverse cardiovascular outcomes, including hypertension, hemorrhagic stroke, and atrial fibrillation, and to various forms of liver disease and pancreatitis [7].

The life expectancy of patients with psychotic disorders is two decades shorter due to the cardiovascular disease that may co-occur with their mental health condition [10]. Other major comorbidities among psychotic patients include prediabetes and diabetes mellitus. When antipsychotic drugs are prescribed, the risk of weight gain, obesity, type 2 diabetes, and sudden cardiac death [11] increases.

The bottom line is that the pathways leading to comorbidity of mental disorders and physical NCD are complex and bi-directional, and care for persons with these conditions needs to be coordinated.

### The NCD Care Agenda and Mental Health Care

Despite the emerging evidence that links mental illness and physical NCD, and the high costs of unaddressed mental illness on society, mental health care is too often left out of discussions on NCD and the global health care agenda.

Without integration of mental health care into the NCD agenda, current NCD initiatives will be less effective and more costly. The comorbidities of mental disorders and NCD are associated with substantial individual and societal health care costs [12]. According to the Agency for Healthcare Research and Quality (AHRQ), the five most costly conditions in the United States between 1996 and 2006 were heart disease, trauma-related disorders, cancer, asthma, and mental disorders, with the largest increase in expenditures being for mental and trauma-related disorders [13]. Care for people with three or more chronic conditions accounts for more than 80% of Medicare health care costs in the United States [5]. According to the 2005 National Claims Database, the presence of comorbid depression or anxiety significantly increased expenditures. The average monthly increase paid by Medicare in 2005 over previous years' expenses for individuals with depression or anxiety was $560 and $710, respectively [14].

NCDs pose a substantial economic burden that will evolve into a staggering one globally over the next 20 years. According to the World Economic Forum, NCD, including cardiovascular disease, chronic respiratory disease, cancer, diabetes and mental disorders, will result in a global cumulative output loss of US $47 trillion over the next two decades [15]. High-income countries currently bear the majority of the economic burden for NCD; however, this burden is expected to be even greater for low- and middle-income countries as their economies and populations grow.

### The Collaborative Care Model

The growing burden of NCD and mental disorders demands new ways of organizing health systems and clinical practices to deal with new challenges. As many as 15 to 30% of all patient referrals for mental health care are made by primary care physicians; mental health care and NCD care should be offered together in primary care platforms [16],[17]. A promising strategy is to use a collaborative care model, which restructures the roles of health care providers and introduces a team-based approach to management of chronic and complex medical conditions. Tasks can be shifted and shared with specialists supporting primary care providers and community health workers to routinely identify patients who need care (case finding); assess risk factors; educate patients about their illnesses, risk factors, and treatment; intervene with a combination of brief evidence-based pharmacological and psychosocial treatments; teach self-management skills; monitor patients' progress and adherence to treatment; and follow-up over the long term.

The effectiveness of collaborative care in improving quality of care and patient outcomes is well established for single conditions, such as depression in primary care settings [18]–[20]. Increasingly, its effectiveness is recognized for the treatment of depression among patients with cancer [21],[22], diabetes [23], and hypertension in high-income countries [24].

Evidence also supports the effectiveness of collaborative care in treating patients with alcohol-related disorders. Screening, brief interventions such as motivational interviewing, and referral are feasible and can be effective in primary care settings for treating individuals who engage in high-risk drinking [25],[26]. In 23 trials, brief (10- to 15-minute) multi-contact interventions among adults receiving behavioral interventions decreased consumption by 3.6 drinks per week from baseline and reduced heavy drinking episodes by 12%. In addition, 11% more adults compared with control participants reported drinking less than the recommended limits over a 12-month period.

Studies of collaborative care for multiple conditions are virtually nonexistent. In one study in the United States, Katon et al evaluated a primary care-based intervention where screening, education, and treatment were provided simultaneously for patients with major depression and poorly controlled diabetes and/or coronary heart disease. Findings at 12 months showed improved glycated hemoglobin, systolic blood pressure, low density lipoprotein (LDL) cholesterol, and depression outcomes as well as reported quality of life and treatment satisfaction [27]. Interestingly, improvements in the primary outcomes in this study were larger than those in other care management trials for single condition depression [18], diabetes [24], and hypertension [28]. Cost-effectiveness analyses showed that small increases in mental health costs in the first year were offset by cost savings in the second year. Cost savings were observed for up to 5 years [29].

Although emerging evidence suggests that collaborative care is also effective in low- and middle-income countries, such as Chile, India, Uganda, Vietnam, South Africa, and Pakistan [30]–[37], the focus of much of this work was on the management of depression in primary care settings rather than on that of comorbidities explicitly. An exception is the successfully implemention in Chile of the Regime of Explicit Health Guarantees (AUGE), a health reform policy to address health inequalities and improve access, quality, opportunity, and financing for priority NCD, including mental disorders (Box 1).

**Box 1. Case Example: The Chilean National Depression Treatment Program**

In September 2004, the flagship program of Chilean health reform, the Regime of Explicit Health Guarantees (AUGE), became law. This program was conceived to tackle the huge inequalities in health services in Chile by explicitly tackling issues related to access, quality, opportunity, and financing [45] for 56 priority health conditions including depression. All citizens have the right to receive timely and appropriate treatment for these conditions from their private or state health providers. The National Depression Treatment Program that resulted from the reform has successfully integrated mental health treatment into primary care platforms across a network of 520 primary care clinics. Following evidence-based clinical guidelines tested in Chile [35], the program is led by psychologists and general practitioners who are supported by specialists to provide pharmacological therapy and psychosocial interventions for diabetes, hypertension, and depression. The depression program guarantees treatment of mild to moderate depression and has grown steadily with more than 200,000 patients receiving treatment every year since 2006 [46].

Research from low- and middle-income countries has expanded the evidence on collaborative care by demonstrating that task-shifting can be extended to lay health workers, particularly when interventions are simplified and proper supervision is provided [38]. A tiered system of supervision between specialists and primary care providers and between primary care providers and community health workers is feasible in resource-constrained settings and can strengthen the capacity of health care settings.

## Challenges and Opportunities

We have a challenge and an opportunity: to embed mental health care services into primary health care platforms globally. Although these services are being integrated into some primary care settings in the United States and other high-income countries, the collaborative care model has not been widely adopted. Low- and middle-income countries face greater challenges because of grossly under-resourced primary care systems and an even weaker mental health infrastructure. Treatment for most mental health conditions is largely provided in large psychiatric hospitals without adequate referral networks in all levels of care and health systems [39]. Limited human resources, lack of training in mental health and NCD care, and fragmentation within the health systems pose significant challenges. However, health care systems in low- and middle-income countries are developing and changing rapidly, creating an opportunity to shape these systems as well as to learn how best to embed mental health services in a variety of different health system environments and socio-cultural contexts [4].

It is now imperative that the mental health and NCD agendas are coordinated to leverage current political and funding commitments, particularly those aimed at reaching the Millennium Development Goals [40]. The recent 2011 United Nations High Level Summit on NCD and the 2012 World Health Assembly focused on the four largely preventable NCD and recognized the need to address the growing burden of mental health conditions. In doing so, they presented an opportunity for increased attention on the inter-relationships among these disorders and coordinated treatments. The board of the Global Alliance for Chronic Diseases (GACD) [41] has included mental health care as one of its mandates, and the United States National Institute of Mental Health (NIMH) has become an integral member of GACD. Fortunately, through these policy efforts, Grand Challenges initiatives [42],[43], and an increasing number of scientific publications on global mental health [17] [30],[44], these issues are being raised and addressed. With international pressure for the World Health Organization to implement a Global Action Plan for Mental Health, the time to promote a coordinated global mental health and NCD agenda could not be more opportune.

## Strategies and Recommendations

### Address the Rise in NCD

The world population continues to grow. The largest increase is expected in individuals over 60 years of age, who are disproportionately affected by NCD. As urbanization accelerates, aging populations encounter risk factors for NCD that are associated with urban lifestyles, such as poor diet, physical inactivity, and tobacco and alcohol use. Health care must respond to the changing global demographics and to the increased risks associated with lifestyle changes in all age groups. Policy interventions such as the “Best Buys” (e.g., taxation of tobacco and alcohol sales) identified by the World Economic Forum could promote healthy environments and reduce vulnerabilities to NCD. Interventions that change risky behaviors related to smoking, nutrition, alcohol, physical activity, and weight control could be implemented in primary care settings, especially for at-risk individuals. Public health education and promotion of health literacy and healthy lifestyles are needed to help individuals understand and modify their risks and to do so early in life. Integrated interventions targeted especially at the aging population could help in the identification and management of multiple chronic diseases.

#### Use Collaborative Care Approaches in Primary Care Settings

Management of chronic disease relies on opportunistic case finding, assessment of risk factors, detection of early disease, identification of high-risk status, combined pharmacological and psychosocial interventions, and long-term follow-up with regular monitoring and promotion of adherence to treatment. Collaborative care models are financially feasible and have the potential to substantially reduce the burden of managing chronic diseases. Many interventions can be managed effectively by non-specialists and lay health care workers who are supported by specialists. Although implemented in a range of settings, collaborative care models are delivered best in primary care settings.

#### Promote Task-sharing and Task-shifting

Most current research focuses on task-shifting from mental health specialists, such as psychiatrists, to physicians and nurses. However, a small but growing literature from low- and middle-income countries suggests that lay health care workers also can be effective, especially when providing screening, psycho-education, and brief behavioral interventions.

A well-established body of research exists on implementation of evidence-based practices by primary care providers in high-income countries, including interpersonal therapy, cognitive behavioral therapy, behavior activation, and problem-solving therapy for management of depression and anxiety conditions and motivational interviewing for alcohol use disorders. Other evidence suggests these same practices, particularly when simplified, can be effectively delivered by health workers with abbreviated training in low- and middle-income countries.

#### Consider the Unique Social, Political, Cultural, and Health System Environments

Input from those who live in the communities where collaborative care interventions are implemented can be invaluable in ensuring that the interventions are appropriate and sustainable within local health systems. Local leaders, health providers, caregivers, and patients can provide information about local needs, risk behaviors, and the availability of resources. Where health providers are scarce and family orientation is strong, the engagement of family and other caregivers may also support patients in making healthier lifestyle choices, adhering to treatment regimes, and better managing their chronic conditions. The use of context-appropriate, community partner strategies can empower communities to leverage local resources and develop local solutions.

#### Increase Funding for Primary and Mental Health Care and for Research

Primary health care and mental health care are generally underfunded around the globe, especially in low- and middle-income countries. In-country investments are needed to demonstrate national ownership, which is key to translating these commitments to real policy and system changes. To meet the challenge of providing integrated mental health and physical NCD care at the primary care level, more investments are needed to strengthen health care systems, to expand the roles of traditional providers to manage multiple chronic diseases, and to train these individuals for those roles.

The evidence for integrating NCD care and mental health care into primary care comes largely from studies in high-income countries focused on patients with co-morbid physical NCD and depression. More research is needed to understand the best strategies for integrating care for chronic illness with care for a wide range of mental health conditions, particularly alcohol use disorders and severe mental illness, and to address the implementation of collaborative care models into settings in low- and middle-income countries.

Efforts to sustain and scale up of efficacious interventions in primary care settings face several challenges. Health workers are already overburdened with many responsibilities, and in many settings, there are not enough resources to regularly supervise lay health workers and to support them with specialist advisors. More research is needed to examine how to best train lay health workers, what tasks can be shifted to what type of provider, coordination of care between NCD and mental health specialists, and what supervision models are needed to support effective implementation particularly in low-resource settings (Box 2).

**Box 2. Recommendations**

1. Integration of the mental health and NCD agendas needs to address both the changing global demographics and the effect of early life exposures on mental health conditions across the lifespan.
2. Inclusion of mental health in NCD care requires primary care-based integrative platforms and collaborative care approaches for management of multiple conditions.
3. Implementation of evidence-based interventions should promote task-shifting and task-sharing activities.
4. The development of these integrated intervention models must take into account differences in social, political, cultural, and health system parameters.
5. Substantial increases are needed in country-level investments in primary and mental health care, as well as funding for research on best approaches for scaling up these services into NCD care management strategies.

## Conclusion

Collaboration is needed between policy makers, practitioners, consumers, public health researchers, development agencies, and funding organizations to develop globally coordinated strategies. However, without country-level recognition and uptake, research and policy guidelines will never translate into action. Economic and health policy makers, including government officials who control health budgets, are often not aware of the links between NCD, mental health, development, and economic growth. These leaders need to be part of the global discourse and the development of local solutions. We must invest in designing health care systems that recognize and address the comorbidity between mental disorders and chronic physical illnesses before we are crippled by the rise in NCD and mental health conditions.

## Abbreviations

NCD: non-communicable chronic diseases
